# Foot drop secondary to rhabdomyolysis: improved foot dorsiflexion and gait after neurolysis and distal nerve transfer—a case series and literature review

**DOI:** 10.1093/jscr/rjad257

**Published:** 2023-05-20

**Authors:** Rahul K Nath, Chandra Somasundaram

**Affiliations:** Texas Nerve and Paralysis Institute, Houston, Texas, USA; Texas Nerve and Paralysis Institute, Houston, Texas, USA

## Abstract

Rhabdomyolysis is a triad syndrome of myalgia, muscle weakness and myoglobinuria due to muscle necrosis. Trauma, exertions, strenuous exercise, infections, metabolic and electrolyte disorders, drug overdoses, toxins and genetic defects are the most common causes of rhabdomyolysis. The etiologies of foot drop are diverse. A few cases of rhabdomyolysis-associated foot drop are reported in the literature. We present five patients with foot drop secondary to rhabdomyolysis; two underwent neurolysis and distal nerve transfer (superficial peroneal nerve to the deep peroneal nerve) surgeries and follow-up evaluations. We found five-foot drop patients secondary to rhabdomyolysis among the 1022-foot drop patients who consulted our clinic since 2004, representing a 0.5% incidence. In two patients, rhabdomyolysis was caused by drug overdose and abuse. In the other three patients, the causes were an assault with a hip injury, a prolonged hospitalization due to multiple illnesses, and an unknown cause with compartment syndrome. Pre-operatively, a 35-year-old male patient had aspiration pneumonia, rhabdomyolysis and foot drop resulting from prolonged ICU hospitalization and a medically induced coma due to a drug overdose. The second patient (a 48-year-old male) had no history of trauma but had a sudden onset of right foot drop after compartment syndrome following the insidious onset of rhabdomyolysis. Both patients had difficulty dorsiflexing their involved foot and walked with a steppage gait before surgery. In addition, the 48-year-old patient had foot slapping while walking. However, both patients had strong plantar flexion (5/5). After 14 and 17 months of surgery, both patients had improved foot dorsiflexion to an MRC grade of 4/5 with an improved gait cycle and walked with no or minimal slapping, respectively. Distal motor nerve transfers in the lower limb facilitate faster recovery and less surgical dissection because of the shorter regeneration distance from the donor axons to the targeted motor end plates through residual neural network connections and descending motor signals.

## INTRODUCTION

Rhabdomyolysis (RML) is a triad syndrome characterized by myalgia, muscle weakness and myoglobinuria, as well as the release of other intracellular muscle components, especially creatinine kinase (CK), electrolytes, into the bloodstream, because of muscle breakdown or necrosis [1–3]. The common complications associated with RML include acute kidney injury or renal failure [4, 5], compartment syndrome [4, 6–11], and in rare cases, peripheral neuropathy [12–17]. The etiology of RML can be a traumatic or non-traumatic muscle injury. The common causes of RML are trauma [18], strenuous exercise [11, 19–23], exertion [9, 21, 23], infections [2], metabolic and electrolyte disorders [2], muscle compression [24], tourniquet [25], immobilization [12, 26], hypothyroidism [27–29], drugs, specifically lipid-lowering drug statin [27–29], toxins [7], drug overdose [4, 30, 31], enzyme deficiency [32], and genetic defects [33, 34].

### Systematic review

We did a PubMed search using the terms rhabdomyolysis and foot drop and retrieved 25 articles. Twenty-one studies were case reports. Twenty-three articles have been published in peer-reviewed English-language scientific journals between 1995 and 2022. Two articles are in non-English, French and German journals; however, the abstract is in English. Eighteen male and three female patients, aged between 17 and 74 years, had a mean and median of 39.2 (SD 17.3) and 34 years, respectively. Five male patients had hypothyroidism, as muscle involvement is a common manifestation of hypothyroidism [27–29]. Among these five patients, two were statin-induced. Drug overdose/abuse and toxic inhalation caused RML and foot drop in four male patients. Prolonged immobilization under the influence of alcohol was reported in one female patient. A young (24-year-old) male patient was recently reported with episodes of rhabdomyolysis and foot drop due to mitochondrial trifunctional protein deficiency (MTP or MTPD) [35]. MTP deficiency is an autosomal recessive fatty acid oxidation disorder caused by HADHA or HADHB gene mutations.

Gait impairment following RML has also been reported in classic heat stroke in elderly patients [36], influenza A H1N1 vaccine administration in one patient [37], Zika virus infection with GBS [38], chemotherapy with Palbociclib treatment in a breast cancer woman [39], and in hypoparathyroidism and hypopituitarism patients [40, 41]. These patients have recovered after the appropriate treatments for kidney injury (hemodialysis), and outpatient physiotherapy [37, 39], except for two elderly patients [36]. One had an ataxic gait, and another patient survived with quadriplegia [36]. There was no report that these patients had surgery for their lower limb defects.

## METHODS AND PATIENTS

### Medical history of five patients with foot drop secondary to RML in our present report

#### Patient 1

Thirty-five years old male patient was in prolonged hospitalization in ICU due to a drug overdose and in a medically induced coma. The patient had aspiration pneumonia and RML. He initially had tingling in his thumb and index finger and weakness in his left arm, which resolved, but he had persistent bilateral foot drop with limited range of motion (ROM) at the ankle-dorsiflexion, weak in dorsiflexion but strong in plantar flexion and toe extension bilaterally.

#### Nerve conduction study, EMG and MRI results of patient 1

MRI of the patient’s calves demonstrated significant abnormality of anterior tibialis muscles consistent with RML and the strength was normal proximally. The right peroneal extensor digitorum brevis (EDB) motor response amplitude was weak and absent in the left. Both the right and left peroneal-TA motor responses were with markedly reduced amplitude. Needle EMG of the selected muscles (L3-S1) in both lower extremities showed no insertional activity and no motor potentials in TA- and peroneus longus muscles.

#### Patient 2

Forty-eight-year-old male patient with no history of trauma or injury but had sudden onset of right foot drop after compartment syndrome of the right lower extremity following the insidious onset of RML.

#### Nerve conduction study and EMG report of patient 2

The right deep peroneal motor nerve and superficial sensory nerves demonstrated no electrical activity indicating an axonal and demyelinating neuropathic process. The right and left lower extremity muscles showed denervation potentials. Fibs/PSWs (+1) indicated resolving muscle tissue breakdown.

#### Patient 3

A thirty-one-year-old female patient with a history of bipolar disorder, intoxicated with psychotropic drugs. Her parents found her unconscious with right leg edema and skin ulcerations consistent with cellulitis, RML and developed foot drop.

#### Patient 4

Twenty-eight-year-old female—an assault led to a massive hip injury resulting in RML and loss of feeling in her left leg, and the patient was wearing AFO.

#### Patient 5

A fifty-three-year-old female patient had a foot drop resulting from hospital admission, which lasted seven weeks. The patient had multiple illnesses simultaneously, including pneumonia, sepsis and RML, caused by damage to the perineal nerve (EMG). The patient’s legs were swollen, as shown in Video 3a. After her initial hospital discharge, she was readmitted with blood clots and a pulmonary embolism. Hence, she was on blood thinners and had a mesh implant, which was expected to stay with her for another two months.

Two patients (1 and 2 on the list) had the following surgery with the senior author (RKN) and follow-up evaluations.

### Surgical procedure

The patient was brought to the operating room and underwent general anesthesia. The involved leg was prepped and draped in a sterile fashion. Pre-operative SSEP electrical testing showed abnormal conduction through the peroneal and tibial nerves on the affected side. The patient was placed in the supine position with a hip bump, and an incision was made in the lateral calf. Immediately upon entering the soft tissue of the fibular neck anatomical area, dense scar and connective tissue were encountered. The superficial and deep peroneal nerves were dissected free and externally neurolyzed. The superficial and deep peroneal nerves underwent internal neurolysis and intraoperative fascicular electrical testing. The deep peroneal nerve was found to have a decreased electrical conduction signal even after neurolysis. All fascicles had zero conduction through the supplied anterior tibialis muscle. The deep branch was proximally neurolyzed to allow sufficient length for the nerve transfer, then transected.

A reasonable fascicle group from the superficial peroneal nerve matched in size to the deep peroneal nerve was selected. A group of nerve fascicles was transected and transposed to the area of the deep peroneal nerve. The donor superficial peroneal nerve fascicle group was sutured to the deep peroneal nerve using 9–0 nylon epineurial sutures placed circumferentially. Therefore, more function could be gained for the tibialis anterior by rerouting some of the motor fibers of the superficial peroneal nerve, which is a relatively short distance for regeneration. The surgery involved meticulous micro neurosurgery. There were no complications, and the wound was closed in two layers with absorbable sutures. The patient tolerated the procedure well and was alert and extubated following surgery. There were no complications, and blood loss was minimal.

We and other investigators have successfully performed distal nerve transfer in the lower limb, as it facilitates faster recovery and less surgical dissection [42, 43] because of the shorter regeneration distance from the donor axons to the targeted motor end plates [44, 45].

We used Microsoft Excel 2013 software for descriptive statistical data analysis.

## RESULTS

Five-foot drop patients secondary to RML were identified among the 1022-foot drop patients who consulted our clinic since 2004 [46], representing a 0.5% incidence. RML was caused by drug overdose and abuse in two patients. The following were the causes for the other three patients in this report:

- an assault with a hip injury
- a prolonged hospitalization because of multiple illnesses
- an unknown cause with compartment syndrome.

Preoperatively, the 35-year-old male patient had difficulty dorsiflexing his involved left foot and had a steppage gait (2/5), as shown in Video 1a. However, he had strong plantar flexion (4/5) and toe extension (4/5). The second patient (a forty-eight-year-old male) could not dorsiflex his involved right foot and walked slowly with a slapping foot (2/5), as shown in Video 2a. After 6 and 17 months of neurolysis and superficial peroneal nerve transfer to the deep peroneal nerve transfer surgery, both patients had improved foot dorsiflexion to an MRC grade of 4/5 with an improved gait cycle, and walked faster compared to pre-surgery, as shown in Videos 1b and 2a.

Our present case series includes three female patients. They did not have surgery; one was on blood thinners and had a mesh implant, which was expected to stay with her for another two months. Another female patient was wearing AFO.

## DISCUSSION

Despite the PubMed search result for rhabdomyolysis being 11 231 on 04 March 2023, the search terms rhabdomyolysis and foot drop retrieved only 25 articles, and twenty were case reports. The terms rhabdomyolysis, gait, and patient retrieved 19 articles, which exclude animal research reports. The incidence of gait impairment and foot drop in RML patients is low, about 0.4%. Most of these patients recovered from RML after hemodialysis for acute kidney injury or renal failure but developed permanent foot drop [4–10, 13, 17, 19, 20, 23–30]. There was no report on these patients who were discharged with permanent foot drop if they had surgery for foot drop.

One of five patients in our case series who underwent neurolysis and peroneal nerve transfer for his left foot drop was pre-operatively hospitalized in ICU due to a drug overdose and in a medically induced coma. The second patient, who also underwent the above surgery for his involved right foot, had compartment syndrome of the right lower extremity following the insidious onset of RML. Acute compartment syndrome (ACS) is characterized by a rise in pressure within a closed fascial space and is non-traumatic [47]. Of 24 PubMed retrieved articles with RML and foot drop, seven-foot drop cases were reported with compartment syndrome (6 males and one female). It has been associated with prolonged immobilization due to alcohol and other drug intoxication (3 patients) [4, 7, 30, 31], high-level athlete (one patient) [11, 19–23], and hypothyroidism (3 patients) [8, 27–29].

Although only three female patients were reported in PubMed literature who had RML with persistent foot drop, our case series has three female patients. However, three are out of 1022-foot drop patients who consulted our clinic since 2004, with an incidence of (0.3%). None of the three female patients had surgery; one was on blood thinners and had a mesh implant, and another female patient was wearing AFO.

Of the five patients in our report, two had drug overdoses; two were hospitalized for a long time; one had multiple illnesses, and another was in a medically induced coma. None of our five patients were under statin medication or with hypothyroidism or exertion induced, although these have been primarily reported [5, 8, 9, 11, 19–23, 27–29]. The RML associated with hypothyroidism is mainly due to statins or vigorous exercise. [27–29]. RML can be a challenge in the postoperative setting. Non-supine position during surgery, tourniquet use, and administration of certain medications, such as suxamethonium, have been also reported as possible risk factors for RML [48–50].

## CONCLUSIONS

Distal motor nerve transfers in the lower limb facilitate faster recovery and less surgical dissection because of the shorter regeneration distance from the donor axons to the targeted motor end plates through residual neural network connections and descending motor signals.

## Supplementary Material

Video_1a_Patient_1_Pre-op_rjad257

Video_1b_Patient_1_Post-op_rjad257

Video_2a_Patient_2_Pre-op_rjad257

Video_2b_Patient_2_Post-op_rjad257

## Data Availability

The data that support the findings of this study are available from the corresponding author, [RKN], upon reasonable request.
